# Construction of a risk prediction model for axillary lymph node metastasis in breast cancer based on gray-scale ultrasound and clinical pathological features

**DOI:** 10.3389/fonc.2024.1415584

**Published:** 2024-11-19

**Authors:** Quifang Ouyang, Qiang Chen, Luting Zhang, Qing Lin, Jinxian Yan, Haibin Sun, Rong Xu

**Affiliations:** ^1^ Ultrasound Department, Second Affiliated Hospital of Fujian University of Traditional Chinese Medicine, Fuzhou, Fujian, China; ^2^ Department of Modern Technology, Fujian Juvenile & Children’s Library, Fuzhou, Fujian, China; ^3^ Key Laboratory of Chinese Medicine Preparation for Medical Institutions in Fujian Province (Fujian University of Traditional Chinese Medicine), Fuzhou, Fujian, China

**Keywords:** ultrasound, radiomics, breast cancer, lymph node, prediction model

## Abstract

**Purpose:**

This study aimed to develop a model to predict the risk of axillary lymph node (ALN) metastasis in breast cancer patients, using gray-scale ultrasound and clinical pathological features.

**Methods:**

A retrospective analysis of 212 breast cancer patients who met the inclusion criteria from January 2011 to December 2021 was carried out. Clinical and pathological characteristics, including age, tumor size, pathological type, molecular subtype, estrogen receptor (ER), progesterone receptor (PR), human epidermal growth factor receptor 2 (HER2), and proliferation cell nuclear antigen (Ki-67), were examined. Preoperative ultrasound examinations were performed, and ultrasound radiomics features of breast cancer lesions were extracted using Pyradiomics software. The data was divided into training (70%) and testing (30%) sets. A predictive model for axillary lymph node metastasis (ALNM) was established by combining clinical and ultrasound features. The diagnostic performance of the model was evaluated using receiver operating characteristic (ROC) curves and five-fold cross-validation.

**Results:**

The rate of lymph node metastasis was 41.51%. Using LASSO algorithm, 17 features linked to ALN metastasis were extracted from a comprehensive databank of 8 clinical features and 1314 ultrasound radiomic attributes. Of these, four were clinical-pathological features (tumor size, tumor type, age, and expression levels of the Ki-67 protein), and 13 were radiomic features. And the following features exhibited both high weights and correlation coefficients: tumor size (R=0.29, weight=0.071), tumor type (R=-0.24, weight=-0.048), wavelet-LH_glcm_Imc1 (R=0.28, weight=0.029363), wavelet-LH_glszm_SZNUN (R=-0.20, weight=-0.028507), and squareroot_ firstorder_ Minimum (R= -0.25, weight= -0.059). The ROC area under the curve for the model in the training and testing sets was 0.882 (95% CI: 0.830-0.935) and 0.853 (95% CI: 0.762-0.945), respectively. The predictive model demonstrated a sensitivity of 87.5% on the training set and 79.2% on the test set, with corresponding specificities of 75.0% and 77.5%, accuracy of 80.4% and 78.1%, respectively. When evaluated using 5-fold cross-validation, the model achieved an average test set area under the curve (AUC) of 0.799 and a training set AUC of 0.852.

**Conclusion:**

The clinical-radiomic model has the potential to predict axillary lymph node metastasis in breast cancer.

## 1 Introduction

Breast cancer remains one of the most prevalent malignancies affecting women worldwide, with metastasis to the axillary lymph nodes (ALN) being a critical determinant of prognosis and treatment strategies (1). Accurate prediction of axillary lymph node metastasis (ALNM) risk is essential for guiding clinical decision-making and optimizing patient outcomes.

The conventional assessment methodologies, which predominantly utilize clinical pathological attributes such as tumor size, grade of differentiation, and receptor status including estrogen receptor (ER), progesterone receptor (PR), human epidermal growth factor receptor 2 (HER2), are insufficient to capture the heterogeneity of individual patients and the complexities inherent to tumor biology (2, 3). Additionally, the assessment of ALN status has relied heavily on invasive procedures, including sentinel lymph node biopsy or axillary lymph node dissection. As a result, these procedures carry inherent risks and limitations, highlighting the need for non-invasive, reliable methods for predicting ALNM risk (4).

Currently, imaging examination is used as a non-invasive method for preoperative confirmation of ALN status, such as mammography, ultrasound, and magnetic resonance imaging. Among the various imaging technologies, ultrasound is the main method for the preoperative assessment of the axilla in women with initial diagnosis of breast cancer due to its cost-effectiveness, simplicity, and high spatial resolution (5). Many studies have shown that ALN morphological features detected by axillary ultrasound are helpful in predicting ALNM. However, the low sensitivity has limited its widespread use. For the majority of early-stage breast cancer patients with clinically negative lymph nodes, axillary ultrasound shows no positive signs, which may lead to false-negative results in early-stage ALNM patients. Therefore, there is an urgent need to explore new methods not based on axillary examination, especially for patients with early-stage breast cancer.

Grayscale ultrasound, an efficacious and non-invasive diagnostic tool, plays a pivotal role in the assessment of breast cancer. It exhibits excellent spatial resolution for visualizing breast tissues. Therefore, combining grayscale ultrasound with clinical pathological features to construct a predictive model for ALNM risk has become an area of intense investigation within oncological diagnostics and treatment.

The emerging field of radiomics, dealing with the quantitative analysis of medical images, offers promising avenues for predicting metastatic involvement (6). This technology converts medical images into quantifiable data that facilitates image reconstruction, denoising, segmentation, and extraction, heralding novel avenues for precise illness characterization (7). Evidently, radiomics has proven effective in predicting lymph node involvement in thyroid carcinoma (8), rectal cancer (9) and gastric cancer (10). Studies by Huang et al. indicate that radiomics surpasses conventional CT in prognosticating lymph node metastasis in colorectal cancer patients (11). Nonetheless, the predictive performance of non-invasive models for ALNM in breast cancer patients remains suboptimal. Previously reported area under the curve (AUC) values for these models range from 0.667 to 0.73 (12, 13), underscoring the necessity for further improvement. Moreover, limited research has addressed the combined influence of primary tumor ultrasound characteristics and clinical parameters on lymph node metastasis (14). Therefore, this study aims to elucidate risk factors for ALNM related to grayscale ultrasonographic properties and the clinicopathological features of the primary breast tumors. Additionally, a support vector machine (SVM) model will be established based on these factors to predict the likelihood of ALNM.

## 2 Methods

### 2.1 General information

This study was approved by the Hospital’s Medical Ethics Committee (SPHFJP-T2022007-01). Individual consent was waived. A retrospective analysis was performed on the preoperative breast ultrasound grayscale images of patients who were examined by the Ultrasound Department of the Second People’s Hospital Affiliated to Fujian University of Traditional Chinese Medicine from January 2011 to December 2021. All patients were diagnosed with primary breast cancer through ultrasound-guided biopsy or subsequent resection.

Inclusion criteria: (1) Confirmed primary breast cancer diagnosis through biopsy or surgical pathology examination; (2) No prior biopsy of breast cancer lesions, or local or systemic treatment targeting breast cancer lesions before breast ultrasound and mammography; (3) Single unilateral lesion; (4) Complete axillary lymph node dissection with comprehensive pathology results. Exclusion criteria: Patients with lesions on ultrasound images that are poorly demarcated, preventing accurate image analysis; patients with missing clinical data or incomplete pathology results; patients with previous breast surgery or other surgical history that could affect the ultrasound results; patients with bilateral breast cancer or multiple lesions within the same breast; patients who did not undergo complete axillary lymph node dissection or lacked corresponding pathology reports.

### 2.2 Ultrasound examination method

The patients were positioned supine, with their hands elevated and positioned on either side of the head, enabling comprehensive exposure of both breasts and axillae. The examination leveraged color Doppler ultrasound diagnostic instruments from Philips, GE, or Siemens, equipped with a high-frequency linear array probe (frequency 9–12 MHz). Upon lesion detection, two-dimensional images were acquired and archived in the ultrasound workstation. Image quality control was performed by two seasoned radiologists, Qing Lin and Luting Zhang. Both radiologists collaborated to ascertain the accuracy and dependability of the findings.

### 2.3 ALN surgical staging and pathological assessment

The surgical approach adheres to relevant guideline protocols. For patients who were candidates for sentinel lymph node biopsy (SLNB), methylene blue was used as the indicator for initial SLNB. If intraoperative frozen section or postoperative paraffin pathology suggested SLN positivity, continue with or subsequently perform ALN dissection. ALN pathology was assessed by intraoperative frozen section and postoperative paraffin section.

The surgical strategy employed in the study strictly followed pertinent medical guidelines. For patients who met the criteria for sentinel lymph node biopsy (SLNB), methylene blue dye was first used for SLNB. If sentinel lymph node (SLN) positivity was indicated by the intraoperative frozen section or post-operative paraffin section examination, an ALN dissection was performed further, or a decision was made whether a second surgery was necessary based on the postoperative pathological results.

### 2.4 Characterization of pathological and clinical data

Eight clinical and pathological parameters were collected as the clinical pathological characteristics of the patients, including: age, tumor size, pathological type (non-special type invasive carcinoma, special type invasive carcinoma, *in situ* carcinoma), molecular typing (Luminal A-type, Luminal B-type, HER2 overexpression type, and triple-negative), estrogen receptor (ER), progesterone receptor (PR), human epidermal growth factor receptor 2 (HER2), and proliferating cell nuclear antigen (Ki-67).

### 2.5 Radiomic analysis

#### 2.5.1 Image annotation

The task of image annotation was jointly undertaken by two experienced sonography physicians. One of them is an attending physician with 5 years of experience, while the other is an associate chief physician with over 12 years of clinical practice. Utilizing ITK-SNAP software, both physicians manually delineated the lesion borders on the maximal cross-sectional images to accurately define the regions of interest (ROIs). Once the annotations were completed, a senior chief sonography physician, with more than 15 years of experience, conducted a thorough review of the annotated ROIs. Based on this review, the optimally qualified ROIs were selected for the extraction of radiomic features. Two sonography physicians independently selected 100 patients to delineate ROIs, with one physician repeating the delineation within one week to ensure consistency. Feature stability was assessed using intra-class correlation coefficients and inter-class correlation coefficient (ICC). Features exhibiting intra-class correlation coefficients and ICC values below 0.75 were omitted from the final dataset.

#### 2.5.2 Feature extraction

The Pyradiomics software version 3.0 was used to extract radiomic features from breast lesion images. For each ultrasound image examined, a total of 1314 features were extracted, covering seven major categories: 252 first order statistical features, 12 shape features, 336 gray level co-occurrence matrix (GLCM) features, 224 gray level size zone matrix (GLSZM) features, 224 gray level run length matrix (GLRLM) features, 196 gray level dependence matrix (GLDM) features, and 70 neighborhood gray tone difference matrix (NGTDM) features (15).

#### 2.5.3 Feature selection

In this study, a total of 1314 radiomics features were extracted from each ultrasound image. After preprocessing and variance filtering, 1205 features were selected. These features were combined with 8 clinical pathological characteristics, yielding a total of 1213 features. All ultrasound features and clinicopathological characteristics were correlated with ALN metastasis using Pearson correlation coefficients. Subsequently, Least Absolute Shrinkage and Selection Operator (LASSO) method was applied for feature selection, and used the selected features for the construction of the radiomics model.

#### 2.5.4 Model establishment

Prior to model building, we divided the dataset into a training set and a test set in a 7:3 ratio. The test set data were not used in model building. Utilizing the filtered features, we adopted Bayesian optimization method to optimize the parameters, in order to select the best-performing parameters that enhance the optimization effect of the model.

#### 2.5.5 Model evaluation

**Figure 1:**
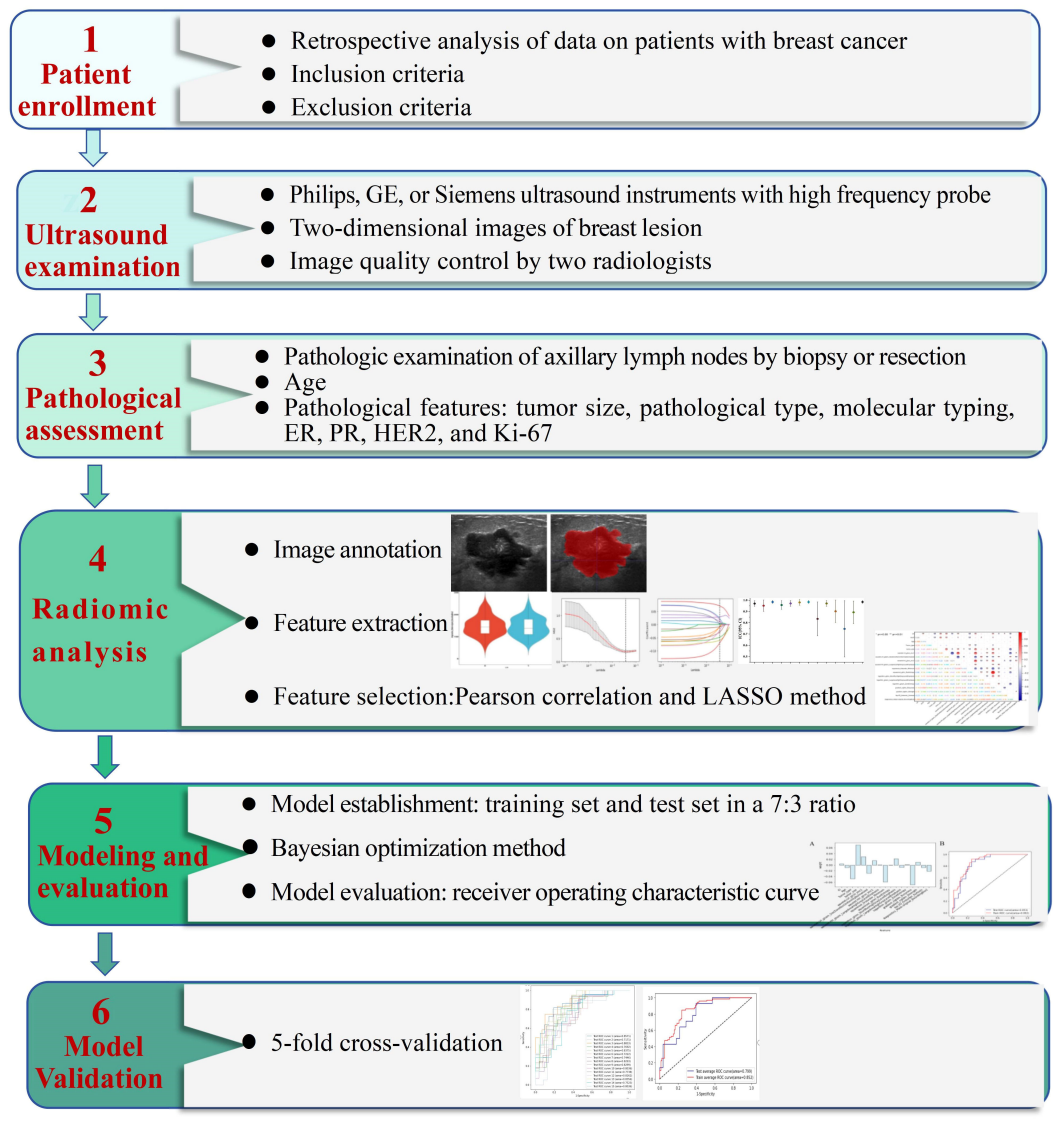
The workflow of this study. ER, estrogen receptor; PR, progesterone receptor; HER2, human epidermal growth factor receptor 2; Ki-67, proliferating cell nuclear antigen; LASSO, least absolute shrinkage and selection operator; ROC, receiver operating characteristic.

To evaluate the model, the pathological results of ALNs were taken as the diagnostic criteria. The receiver operating characteristic curve (ROC) was plotted, and the area under the curve (AUC), along with the 95% confidence interval (CI) was calculated. Based on grayscale ultrasound and clinical pathological characteristics, a predictive model for the risk of axillary lymph node metastasis in breast cancer was constructed. The indexes such as sensitivity, specificity, and accuracy were calculated to assess the diagnostic efficacy of this model in predicting the risk of ALN metastasis in both the training and test sets. In order to accurately assess the performance of the lymph node metastasis prediction model, this study adopted the 5-fold cross-validation strategy. Through 3-round 5-fold cross-validation, the model was trained and tested under various data partitions to ensure the robustness of the results. ROC curve for each round of training and testing was plotted. Finally, the average AUC was taken as the main performance indicator of the model to ensure its generalization ability on new data. The procedure of this study is shown in **Figure 1**.

### 2.6 Statistical analysis

Data analysis was performed using Python software version 3.7.1. Data are presented as mean ± standard deviation. For comparing multiple sample means of quantitative data that are normally distributed and have homogeneity of variance, one-way ANOVA was used for independent events. The least significant difference (LSD) method was employed for pairwise comparisons between multiple sample means that are normally distributed and have equal variances. Data that were normally distributed but had unequal variances were analyzed after transformation or using Dunnett’s test. For data not following a normal distribution, the non-parametric Kruskal-Wallis rank-sum test was used to compare multiple independent sample means. Categorical data were analyzed using the chi-squared (χ^2^) test. A P-value of less than 0.05 was considered to indicate statistically significant differences.

## 3 Results

### 3.1 Clinical pathological characteristics

This study included 212 breast cancer patients, all female, with ages ranging from 25 to 80 years and a mean age of 53.7 years. Among the cases, there were 154 instances of non-special type invasive carcinoma, 31 cases of special type invasive carcinoma, and 27 cases of *in situ* carcinoma. Patients were divided into two groups based on ALNM. ALN metastasis group comprised 88 patients with a mean age of 50 years and non-metastasis group comprised 124 patients with a mean age of 54.5 years. The metastasis rate of lymph node of 41.51%. No statistically significant differences were observed in age between the two groups (t=1.404, P=0.162). Tumor size, pathological type (non-special type invasive carcinoma, special type invasive carcinoma, and *in situ* carcinoma), and levels of Ki-67 were identified as influencing factors for lymph node metastasis. However, there were no significant statistical differences in molecular subtypes (Luminal A, Luminal B, HER2 overexpression, and triple-negative), ER, PR, and HER2 expression between the two groups (**Table 1**).

**Table 1:** Clinical pathological characteristics.

features	metastasis of ALN	non-metastasis of ALN	Value of *t* orχ^2^	*P* value
Age (years)	52.4 + 11.3	54.6 ± 11.3	1.404	0.162
Tumor size			19.279	0.000
Maximum diameter ≤ 2.0 cm	26 (29.5%)	74 (59.7%)		
Maximum diameter 2.1–5.0 cm	53 (60.2%)	45 (36.3%)		
Maximum diameter > 5.0 cm	9 (10.2%)	5 (4.0%)		
Pathological type			16.759	0.008
Invasive ductal carcinoma	75 (85.2%)	79 (63.7%)		
Invasive lobular carcinoma	2 (2.3%)	3 (2.4%)		
Special type invasive carcinoma	10 (11.4%)	21 (16.9%)		
*In situ* carcinoma	1 (1.1%)	21 (16.9%)		
Molecular subtype			8.076	0.445
Luminal A	13 (14.8%)	39 (31.5%)		
Luminal B	46 (52.3%)	50 (40.3%)		
HER2 overexpression	14 (15.9%)	19 (15.3%)		
Triple-negative	15 (17.0%)	16 (12.9%)		
ER			0.546	0.460
Negative	29 (33.0%)	35 (28.2%)		
Positive	59 (67.0%)	89 (71.8%)		
PR			2.531	0.112
Negative	43 (48.9%)	47 (37.9%)		
Positive	45 (51.1%)	77 (62.1%)		
HER2			0.018	0.893
Negative	66 (75.0%)	94 (75.8%)		
Positive	22 (25.0%)	30 (24,2%)		
Ki-67	34.8 ± 25.9	25.3 ± 20.2	3.000	0.003

HER2, human epidermal growth factor receptor 2; ER, estrogen receptor; PR, progesterone receptor.

### 3.2 Feature selection

**Figure 2:**
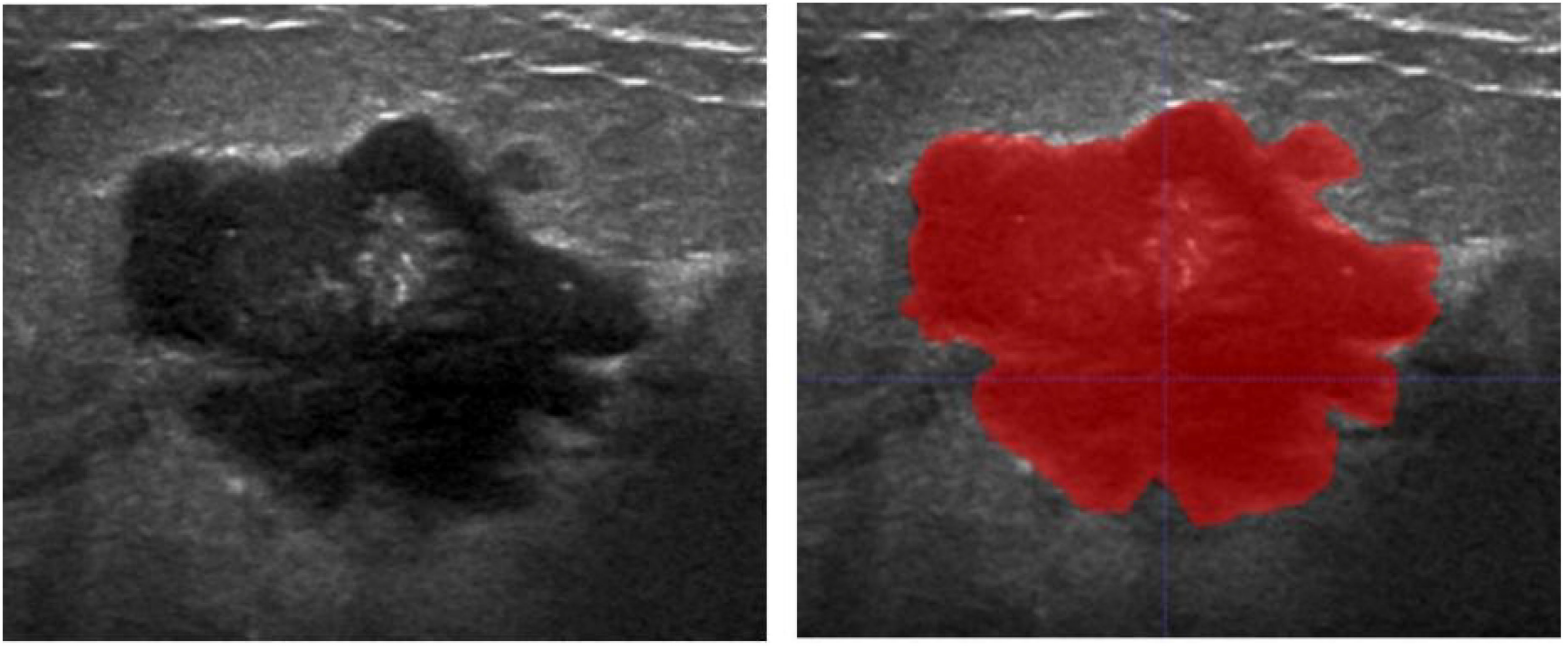
Schematic diagram of conventional ultrasound image of breast cancer lesion (left) and delineation of the region of interest (right).

Utilizing the LASSO algorithm, 17 features linked to axillary lymph node metastasis has been extracted from a comprehensive databank of 1322 clinical and ultrasound radiomic attributes (**Figure 2**). Within this selected group, four features are clinical-pathological, encompassing tumor size, tumor type, age, and expression levels of the Ki-67 protein. The remaining 13 are radiomic features, including variables such as Busyness, squareroot_firstorder_Minimum, informational measure of correlation 1 (Imc1), SizeZoneNonUniformityNormalized (SZNUN), ShortRun HighGrayLevelEmphasis (SRHGLE), BoundingBox2, wavelet-HH_glcm_Imc1, gradient_ngtdm_Strength, Strengthlbp-2D_firstorder_ Kurtosis, logarithm_glszm_ LargeAreaHighGrayLevel Emphasis RunEntropy, ZoneEntropy, and wavelet-HH_glszm_LargeAreaHighGray Level Emphasis (LAHGLE). These 17 discriminative features, comprising both clinical-pathological and radiomic factors, were subsequently saved as labels for further analysis. The 13 features screened in this model exhibit ICC values exceeding 0.75, denoting strong reliability.

### 3.3 Correlation matrix heatmap of selected radiomics features

**Figure 3:**
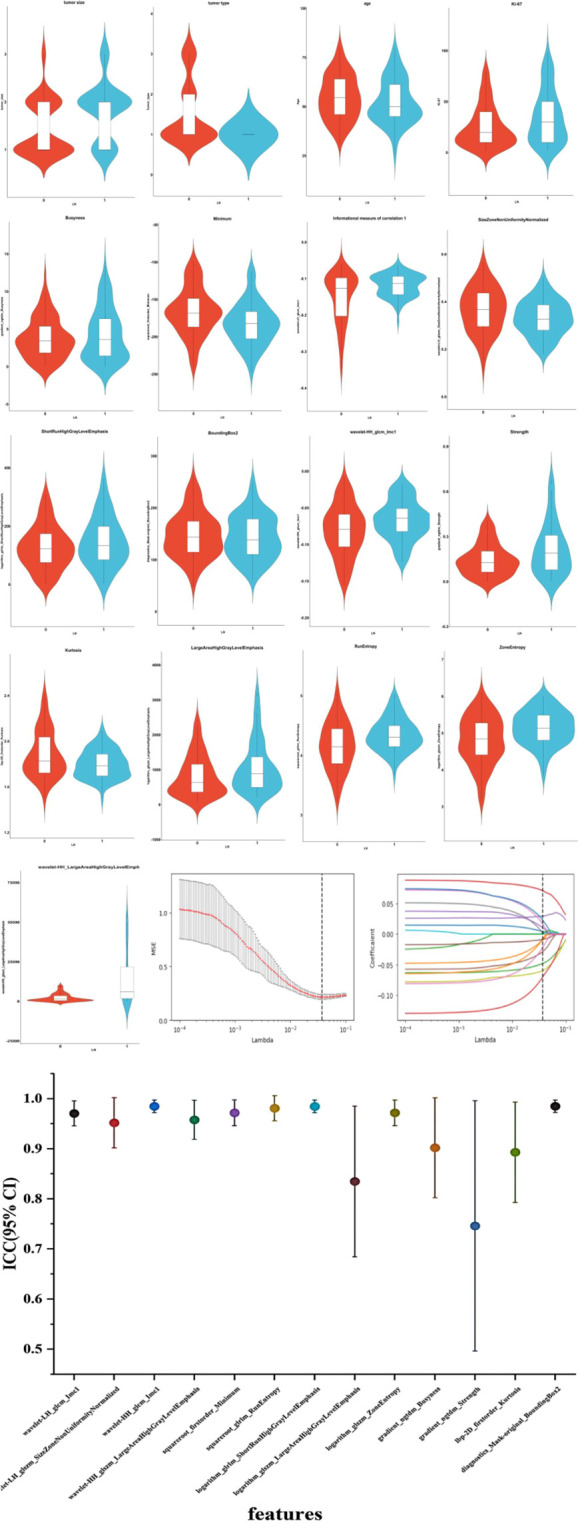
Ultrasound radiomics features associated with lymph node metastasis were selected using LASSO algorithm. And the inter-class correlation coefficient of the key ultrasound features was analyzed. The mean squared error (MSE) graph for radiomic feature selection using LASSO logistic regression; LASSO stands for Least Absolute Shrinkage and Selection Operator; Lambda (λ) represents the LASSO coefficient. The dotted vertical line indicates the optimal lambda value corresponding to the smallest average standard error, which is used for feature selection. The red line represents the average of the MSE. The gray area represents the range of the MSE’s standard deviation. The coefficient distribution graph for the radiomic features is selected by the model. It shows the changes in each feature’s coefficients as the λ value changes, with each line representing a separate radiomic feature. Inter-class correlation coefficient with 95% confidence interval of the key ultrasound features is analyzed. LN, lymph node. 0, non-metastasis of ALN. 1, metastasis of ALN. ICC, inter-class correlation coefficient; CI, confidence interval.

**Figure 4:**
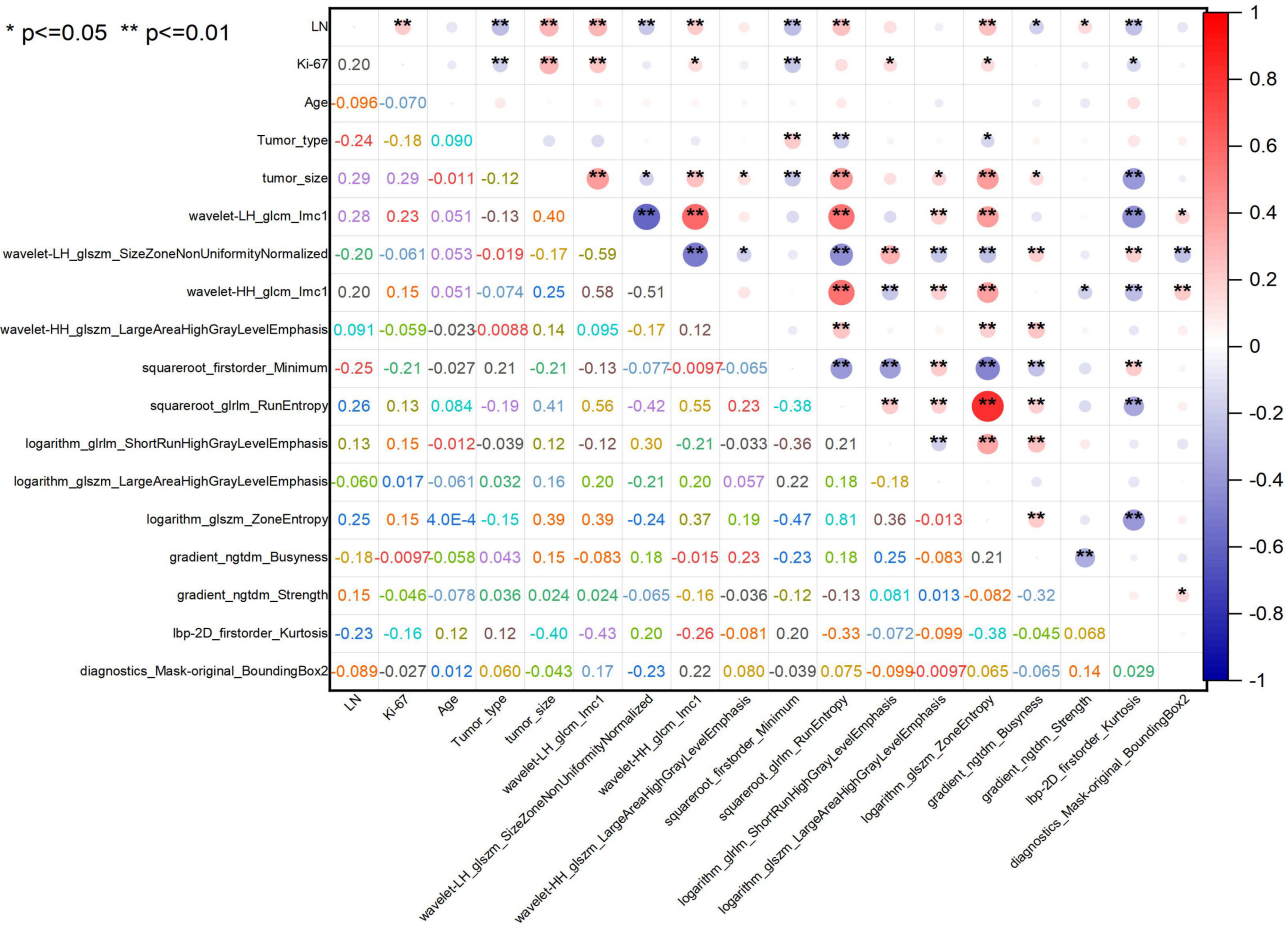
Correlation matrix heatmap of selected radiomics features. The correlation coefficients and magnitude of the correlation between the features. ^*^ *P* ≤ 0.05, ^**^ *P* ≤ 0.01.

The correlation coefficients among the 17 features were generally low, suggesting that the feature clusters were not redundant and that each feature supplied distinct information to the models (**Figure 3**). The correlation coefficients (left) and the corresponding p-values (right) were presented (**Figure 4**). Based on the most significant characteristics identified by the Pearson correlation coefficient, the top ten features were identified, including Ki-67 (R=0.20), Tumor type (R=-0.24), Tumor size (R=0.29), wavelet-LH_glcm_Imc1 (R=0.28), wavelet-LH_glszm_SizeZone NonUniformity Normalized (SZNUN, R=-0.20), wavelet-HH_glcm_Imc1 (R=0.20), squareroot_firstorder_ Minimum (R=-0.25), squareroot_glrlm_RunEntropy (R=0.26), logarithm_glszm_ ZoneEntropy (R=0.25), and lbp-2D_firstorder_Kurtosis (R= -0.23).

### 3.4 Construction and evaluation of support vector machine model

**Figure 5:**
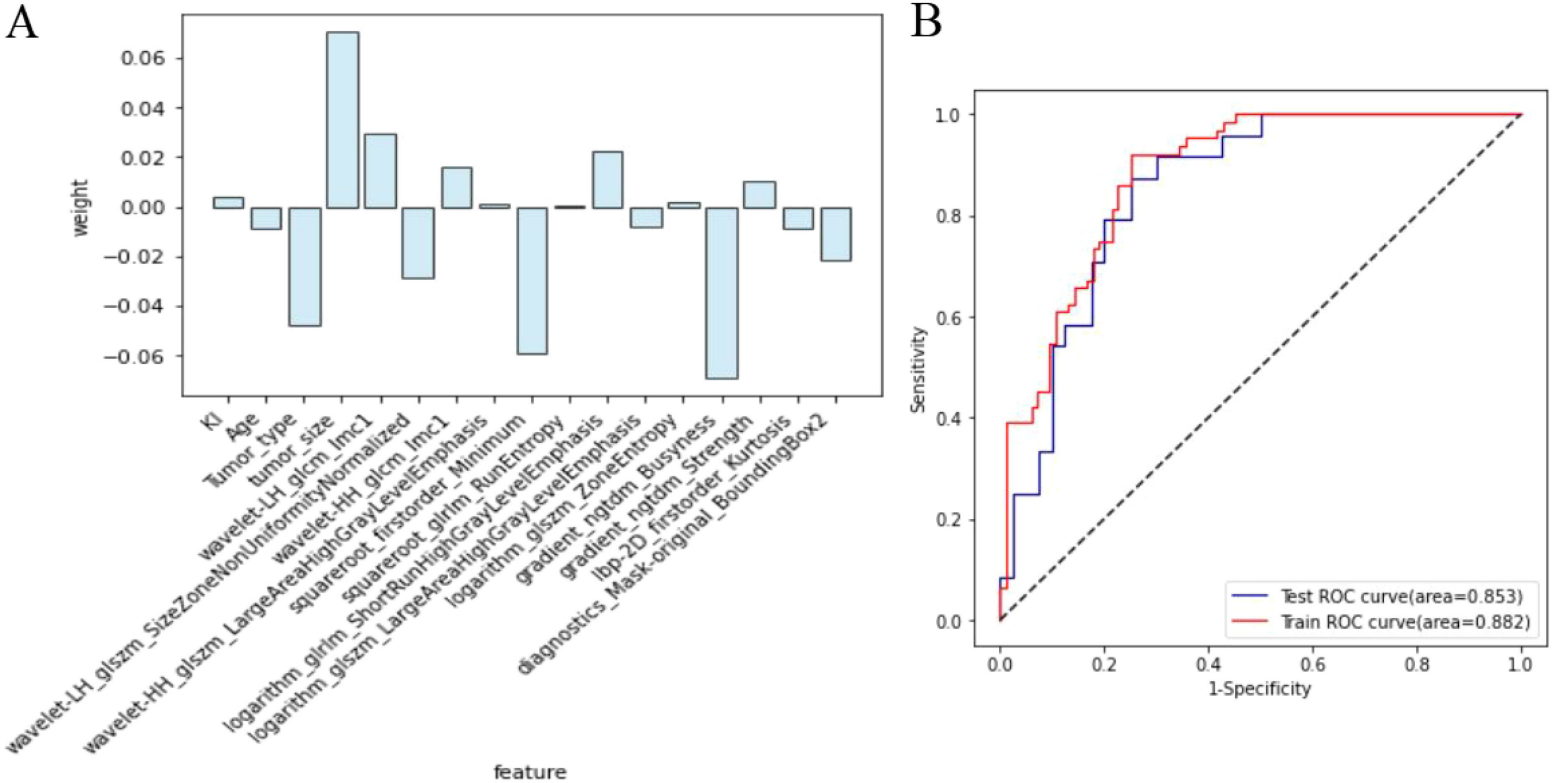
Diagnostic efficacy of the breast cancer lymph node metastasis model. **(A)** The clinicopathologic and radiomic features selected and their respective weights; **(B)** Comparison of the area under the receiver operating characteristic curve for the model in both training set and test set.

The clinical features include tumor size, type, age, and Ki-67 expression, with corresponding weight coefficients of 0.071, -0.048, -0.009, and 0.004, respectively. Among the 13 radiomic features, the top 5 ranked are Busyness, Minimum, Imc1, SZNUN, and SRHGLE, with weight coefficients of -0.069, -0.059, 0.029363, -0.028507, and 0.022331, respectively. Among all the 17 features ranked by weight and correlation coefficients, the following exhibited both high weights and correlation coefficients: tumor size (R=0.29, weight=0.071), tumor type (R=-0.24, weight=-0.048), Imc1 (R=0.28, weight=0.029363), SZNUN(R=-0.20, weight= -0.028507), and Minimum (R=-0.25, weight= -0.059). Utilizing these features, along with clinical-pathological factors, a predictive model for axillary lymph node metastasis was developed employing SVM. The model demonstrated robust predictive ability with areas under the receiver operating characteristic (ROC) curve of 0.882 (95% CI: 0.830-0.935) for the training set and 0.853 (95% CI: 0.762-0.945) for the test set, as illustrated in **Figure 5**. The model’s performance evaluation parameters, including sensitivity, specificity, accuracy, positive predictive value, and negative predictive value, are detailed in **Table 2**.

**Table 2:** Diagnostic efficacy of the support vector machine model in predicting axillary lymph node metastasis.

set	AUC	sensitivity	specificity	accuracy	negative predictive value	positive predictive value
Training Set	0.882	87.5% (56/64)	75.0% (63/84)	80.4% (119/148)	88.7% (63/71)	72.7% (56/77)
Test Set	0.853	79.2% (19/24)	77.5% (31/40)	78.1% (50/64)	86.1% (31/36)	67.9% (19/28)

### 3.5 Model performance validation

**Figure 6:**
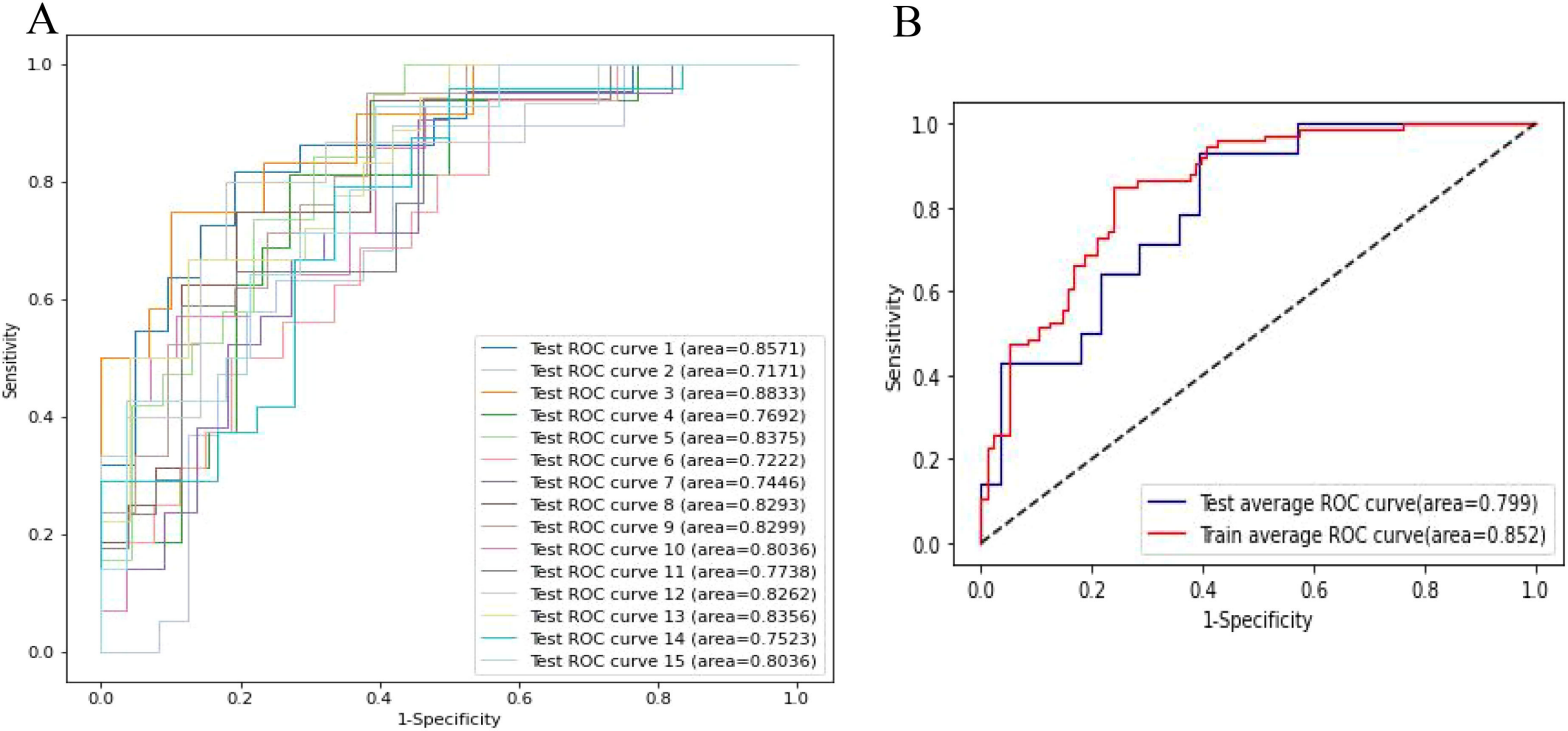
The cross validation of model performance. **(A)** Receiver operator curves (ROC) of the clinical-radiomic model in the test cohort. **(B)** Averaged ROC evaluated by cross-validation.

The lymph node metastasis prediction model, evaluated through 3 rounds of 5-fold cross-validation, showed that the test set AUC ranged from 0.717 to 0.883, with an average AUC of 0.799, demonstrating reliable predictive performance on the test set. The AUC for the training set was 0.852, indicating high model fit during training (**Figure 6**).

## 4 Discussion

Breast cancer, a leading malignancy in women, requires accurate prediction of ALNM to guide treatment strategies and prognosis (16). Radiographic examinations, particularly ultrasound, serve as valuable preoperative tools for the evaluation of lymph nodes and substantially guide clinicians towards optimal treatment strategies. Nevertheless, the sensitivity of ultrasound for detecting early-stage ALN metastasis is notably limited, which underscores the necessity for the development of more efficacious diagnostic techniques. In this study, we identified tumor type, tumor size, Imc1, SZNUN, and Minimum as important risk factors for ALNM. Moreover, we constructed a SVM model to predicate ALNM based on ultrasound findings and clinicopathological characteristics, which demonstrated excellent performance, as evidenced by AUC values of 0.882 (95% CI: 0.830-0.935) for the training set and 0.853 (95% CI: 0.762-0.945) for the test set. After cross validation, our model achieved an AUC of 0.799 for the test set and 0.852 for the training set, outperforming previous publications, which recorded an AUC of 0.67 (17).

Our research innovatively delineated critical ultrasound radiomics features linked to ALNM and established a model that integrated clinicopathological indicators and ultrasound features. By leveraging artificial intelligence to improve diagnostic precision, this study makes a substantial contribution to the evolution of personalized medicine and precision oncology of breast cancer.

In the predictive model, tumor size (R=0.29, weight=0.071) and tumor type (R=-0.24, weight=-0.048) exhibited significant correlation coefficients and were assigned substantial weights, underscoring their importance as clinical variables. These findings imply a heightened risk of lymph node metastasis associated with larger breast cancer masses and nonspecific types of invasive cancer. These observations are consonant with those reported by Min S K et al., which demonstrate a significant relationship between tumor size and type and the incidence of lymph node metastasis (18). The underlying reason is that an increased tumor area often represents a greater tumor burden, which in turn raises the likelihood of penetration into adjacent tissues, lymphatic channels, and blood vessels. Among invasive cancers, nonspecific types such as invasive ductal carcinoma and invasive lobular carcinoma are known for their aggressive invasiveness and metastatic potential.

Ultrasound features of Imc1 (R=0.28, weight=0.029363), SZNUN (R=-0.20, weight= -0.028507), and Minimum (R=-0.25, weight= -0.059) were also an important predictor factor in ALNM. Imc1 quantifies the complexity of the texture in the image by assessing how every pixel is correlated with its neighbors over the entire image. Tumors with more heterogeneous appearances may have higher Imc1 values (19). The positive correlation between lymph node metastasis risk and Imc1 can be explained by the fact that more aggressive tumors tend to exhibit more complex and heterogeneous patterns on imaging. Imc1 is a predictor of metastatic spread (20). SZNUN measures the variability of size zone volumes throughout the image. Higher SZNUN values correspond to a more variation of zone sizes, whereas lower values indicate more homogeneous distribution. SZNUN feature was mainly associated with nuclear division and cell-substrate junction (21). It is also reported that SZNUN was correlated with the gene expression of Kirsten Rat Sarcoma Viral Oncogene Homolog (KRAS), Signal Transducer and Activator of Transcription 1(STAT1), and Mitogen-Activated Protein Kinase 1(MAPK1) (22). Squareroot_firstorder_Minimum refers to the square root of the minimum pixel or voxel intensity value within a region of interest (ROI) in a medical image. Minimum characterizes the darkest regions (areas with lower intensity values) in the imaging study, which could relate to necrosis. The negative correlation between Minimum and the risk of lymph node metastasis suggests that lower pre-transformed minimum intensity values (which correspond to darker areas on the image) are associated with increased likelihood of metastasis. This could be due to aggressive tumor regions which might be prone to metastasize (23).

The predictive model examined in this study, which incorporates 17 features, has demonstrated remarkable diagnostic efficacy for ALNM in breast cancer. Specifically, it achieved an area under the receiver operating characteristic curve of 0.882 for the training set and 0.853 for the test set. This combined-method model surpasses those relying solely on clinicopathologic factors or ultrasound features in predicting ALNM (24). Moreover, our findings differ from those of Xiong J et al., who developed a nomogram to predict the probability of ALNM (13). Their AUC was 0.705 for the training set and 0.745 for the validation set, slightly lower than the AUC observed in our study. Previous studies have successfully predicted the LN status in breast cancer patients. Sun et al. applied a convolutional neural network to predict axillary lymph node metastasis using primary breast cancer ultrasound images, achieving an AUC of 0.72 (25), which is lower than the performance of our model. This difference may be due to our radiomic approach, which integrates clinicopathological features, enhancing the overall predictive accuracy. Similarly, Fanizzi et al. (26) highlighted the importance of combining histopathological features with other types of data to enhance predictive precision. In line with this approach, we integrated clinicopathological data with radiomic features in our model. Bove et al. (27) developed a predictive model by combining clinical and radiomic features, reporting an AUC of 88.6%. However, they did not extend to a detailed analysis of specific ultrasonographic features. Our method, which uses LASSO for feature selection, aligns with theirs, allowing for precise feature selection that enhances both the interpretability and performance of the model.

In this study, the LASSO algorithm combined with 5-fold cross-validation was employed for feature selection, followed by 3-round 5-fold cross-validation to evaluate the model’s performance. The results demonstrate that the model maintained stable performance across multiple data splits, with minimal fluctuation in the test set AUC and an average AUC of 0.799, indicating strong predictive ability for lymph node metastasis risk. Compared to a single train-test split, cross-validation effectively reduced the risk of overfitting and enhanced the robustness and generalizability of the model.

Notably, we also found that features such as Ki-67, RunEntropy, and ZoneEntropy have high correlation coefficients but low weights, whereas other indicators such as Busyness, SRHGLE, and BoundingBox2 have lower correlations but higher weights. This indicates both connections and differences between correlation coefficients and weight coefficients. Correlation coefficient reflects the degree of linear or nonlinear relationship between features and the target (28). And correlation metrics usually do not incorporate inter-feature relationships but independently assess the association of each feature with the target. Weight coefficient represents the importance of each feature in classification or regression tasks. And its weights reflect the contribution of each feature to separating different categories or predicting the target variable (29). The indicators with high weights but low correlations may due to complex interactions between features, multicollinearity or overfitting. The reason why some indicators exhibit high correlation but low weights can be attributed to factors such as redundancy, noise, and etc.

Nevertheless, we should acknowledge several limitations in our study. Firstly, Since blood flow signals are susceptible to instrument and operator. We excluded this indicator from the construction of the model. Secondly, our study still faces certain technical challenges, such as the quality and interpretability of grayscale ultrasound images, which could impact the accuracy and stability of the model. Additionally, our research lacks external validation from other datasets, warranting further multicenter, large-sample studies to validate the reliability and effectiveness of the model. Future research directions include, but are not limited to, the following: further optimizing the methodology for constructing predictive models, exploring additional combinations of imaging and clinicopathological features to improve model accuracy and stability; conducting multicenter, large-sample external validation studies to assess the generalizability and applicability of this model; and investigating other factors influencing breast cancer metastasis, such as gene expression and microenvironmental factors, to refine risk prediction models and offer comprehensive decision support for clinical practice.

## 5 Conclusions

Our findings underscore the significance of features such as tumor size and type, as well as advanced radiomic metrics (e.g., Imc1, SZNUN, Minimum) in predicting metastatic risk. This study successfully established a model for predicting lymph node metastasis risk by analyzing the clinical and ultrasound radiomics features of breast cancer patients. Our combined predictive model provides more comprehensive and precise diagnostic information, enhancing the potential for personalized treatment.

## Data Availability

The original contributions presented in the study are included in the article/**Supplementary Material**. Further inquiries can be directed to the corresponding author.
